# Hypermethylation of *SOX2* Promoter in Endometrial Carcinogenesis

**DOI:** 10.1155/2010/682504

**Published:** 2010-08-09

**Authors:** Oscar Gee-Wan Wong, Zhen Huo, Michelle Kwan-Yee Siu, HuiJuan Zhang, LiLi Jiang, Ester Shuk-Ying Wong, Annie Nga-Yin Cheung

**Affiliations:** ^1^Department of Pathology, Queen Mary Hospital, The University of Hong Kong, Pokfulam Road, Hong Kong; ^2^Department of Pathology, Peking Union Medical College Hospital, CAMS and PUMC, Beijing 100730, China

## Abstract

This paper aimed at investigating the expression and methylation profiles of *SOX2*, a gene coding for the stem cell-related transcription factor *SOX2*, in endometrial carcinomas. By methylation-specific polymerase chain reaction (MS-PCR), the methylation status of *SOX2* promoter region in 72 endometrial carcinomas and 12 normal endometrial samples was examined. Methylated allele was found in 37.5% (27/72) of endometrial carcinomas but only in 8.3% (1/12) of normal endometrial, significantly more frequent in cancers (*P* = .0472). *SOX2* mRNA level was significantly reduced in endometrial carcinoma compared with nonneoplastic endometrium (*P* = .045). A significant correlation between *SOX2* mRNA expression and hypermethylation of *SOX2* was found (*P* = .024). Hypermethylation of *SOX2* tended to be more frequently found in type II serous or clear cell adenocarcinoma. *SOX2* methylation was also significantly correlated with shorter survival of patients (*P* = .046). In conclusion, epigenetic mechanisms may play a crucial role on the transcriptional regulation of *SOX2* and loss of *SOX2* expression may be related to endometrial carcinogenesis.

## 1. Introduction

Endometrial cancer is the most common cancer found in the female genital tract worldwide [1]. Although endometrial cancers generally show favorable prognosis, the incidence is on the rising trend in North America, Europe, and Asia [2, 3]. There are two major types of endometrial carcinomas exhibiting different histopathology, cell biology, clinical course, and underling genetic alterations [4]. Approximately 70–80% endometrial cancers show endometrioid differentiation and were designated as Type I carcinomas. They are often preceded by premalignant endometrial hyperplasia, which is presumably caused by long-duration unopposed oestrogenic stimulation. Type I carcinomas generally have favorable outcome. Common genetic changes of Type I carcinomas include mutations of K-RAS and PTEN genes, microsatellite instability (MSI) and alteration of beta-catenin [4]. Type II carcinomas are poorly differentiated. In contrast to Type I carcinomas, these tumors are not oestrogen driven and often arise in a background of atrophic endometrium. Type II carcinomas also exhibit a more aggressive clinical course and poorer prognosis than Type I carcinomas. Common genetic changes include mutations of TP53 and CDH1 (E-cadherin) genes [4]. Despite the recent advances in molecular diagnostics, the most important factors in predicting patient prognosis remain to be tumor grade, stage, and subtypes [5, 6].

Sox proteins are transcription factors related by a 79-amino acid high-mobility-group (HMG) DNA-binding domain that was first identified in the mammalian Sry protein [7]. They take up various roles in neural development, including neural stem cell maintenance, glial specification, and lineage-specific terminal differentiation [8]. More than 20 members of the *SOX* gene family have been identified in mammals [9]. Among them, *SOX2* was first found crucial for maintaining the stemness of neural stem cells and then of embryonic stem cells. In conjunction with OCT3/4 and NANOG, *SOX2* is considered a master regulator of mammalian embryogenesis and part of a complex network of transcription factors that affects both pluripotency and differentiation in embryonic stem cells [10]. In fact, forced expression of OCT3/4, *SOX2*, c-MYC, and KLF4 was sufficient to induce stem cell-like pluripotency in adult fibroblast [11] and CD34^+^ blood cell [12].

*SOX2* is dysregulated in many human cancers but its role may vary in different kinds of malignancy. *SOX2* was found to be frequently downregulated in intestinal metaplasia of stomach [13] and gastric cancers [14]. Ectopic overexpression of *SOX2* could inhibit cell growth through cell-cycle arrest and apoptosis in gastric epithelial cells [14]. In contrast, *SOX2* and *OCT3/4* were overexpressed in esophageal squamous cancer and significantly associated with higher histological grade and poorer clinical survival [15]. *SOX2* overexpression was also observed in small cell lung cancer [16], basal cell-like breast carcinomas [17], and glioma [18]. Overexpressed *SOX2* may promote cell proliferation and tumorigenesis of breast cancer cells through enhancing the G1/S transition of cell cycle [19]. Similarly, silencing *SOX2* in glioblastoma tumor-initiating cells leads to stop of proliferation and loss of tumorigenicity [20]. Recently, our team was the first to report loss of *SOX2* and hypermethylation in the promoter region of *SOX2* in trophoblastic diseases including hydatidiform mole and choriocarcinoma [21].

CpG island hypermethylation is a common event in the development of the gynecologic cancers [22]. Our team has previously demonstrated the hypermethylation of RAS-related genes in endometrial carcinomas in association with distinct clinicopathological parameters [23]. To the best of our knowledge, there is no report on the methylation status of *SOX2* gene in endometrial cancers. Therefore, we decided to study the methylation and expression status of *SOX2* in endometrial carcinomas.

## 2. Meterials and Methods

### 2.1. Clinical Samples

Formalin-fixed, paraffin-embedded tissues of 57 cases and frozen tissues of 15 cases of endometrial carcinomas were retrieved for methylation study and mRNA expression analysis. 12 cases of normal endometrium were retrieved for methylation study. In 23 of the 57 carcinoma cases being studied, their corresponding nonneoplastic endometrium was retrieved for mRNA expression analysis. All specimens of tissues were collected at the Department of Pathology, Queen Mary Hospital, The University of Hong Kong. Prior to DNA and RNA extraction, haematoxylin, and eosin-stained section was reviewed to confirm histological diagnosis and purity of the sample. Only samples with more than 75% cancer cells were used.

### 2.2. DNA Extraction and Bisulphite Modification of Genomic DNA

Genomic DNA was isolated from paraffin-embedded tissue by phenol-chloroform extraction after protease K digestion. Conversion of unmethylated cytosine residues in the genomic DNA to uracil by sodium bisulphite was performed as described previously in [24]. 5 *μ*g of DNA was used in the sodium bisulphate conversion. The QIAEX II kit (QIAGEN) was used to purify the converted DNA according to the manufacturer's instructions.

### 2.3. Methylation-Specific Polymerase Chain Reaction (MS-PCR)

The methylation and unmethylation-sensitive primers used in this study have been described previously [21] and were shown in Table 1. The primers amplify a CpG-island located at about 500 bp upstream to the transcription start site of *SOX2* (nm_0003106) [21]. 1.5 *μ*l of bisulfite-converted DNA was amplified in a 25 *μ*l reaction mixture containing 200 *μ*M dNTPs, 10X reaction buffer, 2.5 mM MgCL_2_, 10 pM forward and reverse primers, and 1 U of FastTaq (Roche). Bisulfite-converted normal lymphocyte DNA methylated *in vitro* with Sssl methyltransferase was used as positive control while water was used as no-template controls. The MS-PCR was conducted as following: predenatured for 4 min at 94°C, then at 94°C for 30 seconds, 55°C for 30 seconds, 72°C for 30 seconds for 40 cycles, and finally a 10-min extension at 72°C. Polymerase chain reaction products were separated on 2% Tris-borate EDTA agarose gels, stained with ethidium bromide, and visualized under a UA transilluminator. Cases detected with the presence of methylated alleles were repeated once for confirmation.

### 2.4. RNA Extraction and cDNA Synthesis

RNA was isolated from paraffin-embedded tissue by TRIZOL (Invitrogen) according to the manufacturer's instructions. First-strand cDNA was synthesized from 2.5 *μ*g total RNA with oligo-dTprimer and SuperScript III reverse transcriptase (Invitrogen) according to the manufacturer's instructions.

### 2.5. Quantitative Real-Time Reverse Transcriptase-Polymerase Chain Reaction

The mRNA expression of *SOX2* was investigated using quantitative real-time reverse transcriptase-polymerase chain reaction (RT-PCR). Primers were designed specific to the *SOX2* gene. Prime sequences for *SOX2* and GAPDH (as internal control) are listed in Table 2. Quantitative real-time RT-PCR was performed in a 10 *μ*l reaction, which included 1 *μ*l of cDNA template, 10 pM of each forward and reverse primer, and 5 *μ*l iTaq SYBR Green Supermix with Rox (Bio-rad). Each PCR reaction was optimized to ensure that a single PCR product was amplified and no product corresponding to prime-dimer pairs was present. PCR reactions of each template were performed in duplicate in one 96-well plate. The thermal cycling conditions comprised an initial denaturation step at 95°C for 10 min and 40 cycles at 95°C for 15 sec, and 58°C for 1 min. The expression of *SOX2* was normalized with respect to that of GAPDH. 

### 2.6. Immunohistochemistry

Immunohistochemistry was performed as previously described in [25]. Paraffin sections 4 *μ*m thick was deparaffinized followed by antigen retrieval using microwave treatment. Immunohistochemistry was performed using the streptavidin-biotin complex immunoperoxidase method (Dako, Glostrup, Denmark). Monoclonal primary antibodies for estrogen receptor (ER) (Dako) and progestogen receptor (PR) (Zymed Laboratories, San Francisco, CA) were applied, both at 1 : 150 dilution, and incubated overnight at 4°C. A case of breast cancer was used as positive control in each batch of experiment. Negative control was prepared by replacing the primary antibody with Tris-buffered saline. Assessment of immunoreactivity was performed independently by two pathologists according to percentage of immunor active nuclei: 1: 1–25%; 2: 26%–50%; 3: 51–75%; 4: 76–100%.

### 2.7. Statistical Analyses

Statistical analysis was performed using the Statistical Package Service Solution software (SPSS version 16. 0). The association between methylation status and clinicopathological parameters was tested by chi-square test. The association between methylation status and mRNA expression level was analyzed using Spearman correlation test. For mRNA quantitative analysis, the relative gene expression between groups was compared with unpaired *t*-test (Mann-Whitney test). The association between methylation status and ER/PR immuno-scores was analyzed using Pearson correlation test. *P* values less than.05 were considered statistically significant with two-tailed test.

## 3. Results

### 3.1. Promoter Region of *SOX2* Is Hypermethylated in Endometrial Carcinoma

**Figure 1:**
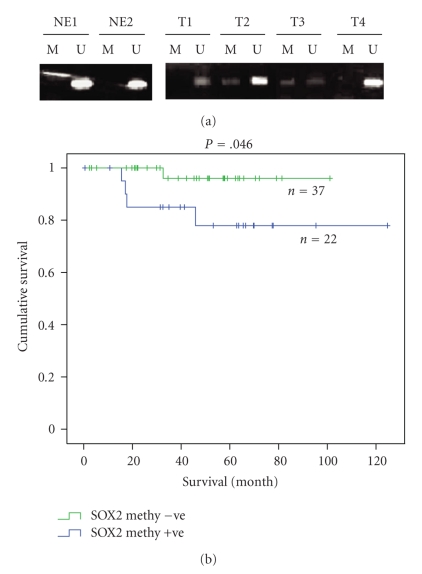
(a) Representative examples of methylation-specific PCR on *SOX2* in endometrial carcinomas (T), and in normal endometrial tissue (NE), demonstrating methylated (M) and unmethylated (U) alleles. (b) Survival curves of patients classified according to the presence or absence of methylated *SOX2* allele.

In a previous study of methylation status of *SOX2* in gestational trophoblastic diseases, we identified a CpG island upstream of the transcription start site of *SOX2* [21]. The methylation frequency of this CpG island in 72 cases of endometrial carcinoma and 12 cases of normal endometrium was assessed by MS-PCR. Hypermethylation of the *SOX2* promoter was observed in 37.5% (27/72) of endometrial carcinomas, and 8.3% (1/12) of normal endometrial tissues (Table 3 and Figure 1(a)). Therefore, more frequent hypermethylation in *SOX2* promoter in endometrial cancers than in normal endometrial tissues was observed (*P* = .0472, chi-square test; Table 3). Moreover, when the cancer samples were grouped according to their histological subtypes, we observed a trend of more frequent *SOX2* promoter hypermethylation in type II (serous and clear cell subtypes) (8/14, 57.1%) than in type I cancers (endometrial subtype) (16/48, 33.3%) though statistical significance was not reached (*P* = .108; Table 4). No correlation was observed, however, between methylation status with histological grade/ stage/ myometrial invasion/ vascular invasion/ age (Table 4).

*SOX2* methylation status correlated with PR expression (Pearson correlation 0.377, *P* = .033) but not with ER expression. Kaplan-Meier analysis also demonstrated a significant correlation between *SOX2* methylation and shorter overall survival (Figure 1(b); *P* = .046, log-rank test).

### 3.2. *SOX2* mRNA Expression Is Lower in Endometrial Carcinomas Than in Their Normal Counter Parts and Is Correlated with the Methylation Status in Carcinomas

**Figure 2:**
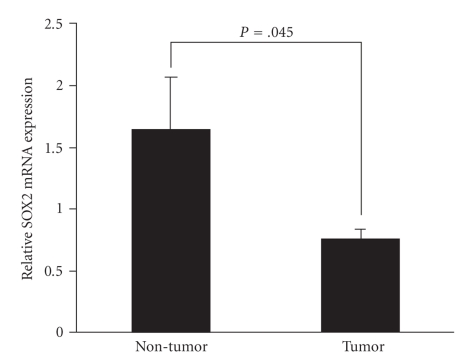
Relative *SOX2* expression in endometrial carcinomas and in normal endometrial tissue.

Out of the 72 cases of endometrial carcinomas tested with MS-PCR, 23 cases have corresponding nonneoplastic endometrium available. As shown in Figure 2, *SOX2* mRNA level was significantly reduced in endometrial carcinoma compared with normal tissues of the same patients (*P* = .045 Mann-Whitney U test; Figure 2). Moreover, there was a significant correlation between *SOX2* mRNA expression and hypermethylation of *SOX2* in endometrial carcinomas samples (Spearman correlation coefficient = 0.470, *P* = .024). 

## 4. Discussion

In this study we tried to answer the question whether the stemness-related transcription factor gene *SOX2* expression is affected by promoter methylation in endometrial cancer. Epigenetic gene silencing through DNA methylation has been suggested to be one of the important steps during endometrial carcinogenesis [23, 24, 26–28]. Promoter hypermethylation of RASSF1A, metallothionein 1E, and related tumor-suppressor genes have been found to correlate with clinicopathological parameters in endometrial cancer [23, 26, 28]. On the other hand, hypomethylation is also found to be important in regulating the expression of the S100A4 gene in endometrial cancer [27]. Here, our results suggest more frequent hypermethylation events, at least in the investigated CpG islands of *SOX2* gene, in endometrial cancer samples than in normal endometrium. Moreover, hypermethylation of *SOX2* promoter was correspondingly matched by a decrease of *SOX2* mRNA level in the samples. Moreover, analysis on patients' survival also linked hypermethylation of *SOX2* with worse clinical outcome. Taken together, our findings support the possibility that *SOX2* gene hypermethylation and downregulation contributes to endometrial carcinogenesis.

Notably, the frequency of hypermethylation in cancer samples was not high (37.5%). This may suggests that other genetic or epigenetic events other than *SOX2* downregulation contribute to endometrial carcinogenesis. Moreover, hypermethylation is a dynamic process. It may exist in early stages of endometrial carcinogenesis such as the precursor lesions and may have reverted to unmethylated state by the time carcinoma is developed. It is also possible that *SOX2* was downregulated by hypermethylation of other CpG islands in the promoter regions of *SOX2* that were not tested in this study. In fact, the CpG island investigated in this study lies at about 200 bp upstream of the transcription start site [21] and CpG islands further upstream may exist (CpG search analysis, data not shown). It is our next aim to study the methylation pattern in CpG islands further away from the transcription start site.

It is interesting to note that, among all clinicopathological parameters examined, hypermethylation of *SOX2* promoter was linked marginally to histologic subtypes, being relatively more common in type II serous and clear cell adenocarcinomas. This finding further supports the notion that type I and type II endometrial cancers represent two different malignancies with different pathologically courses [4]. Indeed, we have reported earlier the significantly more frequent RASSF1A hypermethylation in type I endometrioid carcinomas when compared with the type II carcinomas [23]. 

There was another interesting observation that *SOX2* methylation was weakly correlated with PR expression. Progesterone deficiency relative to estrogen level has been considered as a risk factor for endometrial cancer [29]. High PR expression is usually considered as a good prognostic marker for endometrial cancer [30]. It is hence intriguing that *SOX2* methylation was found to correlate with shorter survival (Figure 1(b)) but also with higher PR level. It is possible that other mechanisms related to hypermethylation of *SOX2* may contribute to poor survival independent of hormonal effects by surmounting the beneficial effect of PR. For instance, suppression of *SOX2* has been reported to facilitate overcoming cell-cycle arrest and apoptosis [14]. Moreover, there are at least two distinct functional isoforms of PR, PR-A, and PR-B, which are derived from the same gene through alternative transcription start sites [31]. It has been shown in mice that PR-B, in the absence of PR-A, actually promotes cell proliferation in the presence of estrogen alone or estrogen and progesterone simultaneously [32]. It is therefore imperative to distinguish PR-A and PR-B in human immunohistochemical studies. In fact, in a recent immunohistochemical investigation conducted in 315 endometrioid endometrial cancer patients, a ratio of PR-A/PR-B < 1 was associated with shorter survival, suggesting PR-B may correlate with poor prognosis [33]. It is important to further our investigation on the relationship between *SOX2* methylation and the statuses of both PR isoforms.

It is currently unclear why *SOX2*, a transcription factor important for self-renewal and pluripotency of stem cells, is downregulated in endometrial cancers. In fact, a meta-analysis of publicly available gene expression data suggested that at least one of the four pluripotency factors Oct3/4, *SOX2*, Klf4, and c-myc is overexpressed in 18 out of 40 cancer types [34]. It was argued that overexpression of the four factors may contribute to the pathological self-renewal characteristics of cancer stem cells. However, overexpression of *SOX2* was not observed in endometrial cancer in the analysis [34]. Our observation that *SOX2* was downregulated in endometrial cancer actually concurs with the mentioned analysis. Moreover, *SOX2* downregulation has been found to be frequent in clinical samples, cancer cell lines and primary cultures of human cancers such as choriocarcinomas [21] and gastric cancer [14]. In choriocarcinoma cell lines, *SOX2* expression is restored following treatment to 5-Aza-2′-deoxycytidine and/or Trichostatin A, demethylation and histone deacetylase inhibitors respectively, and the effect was synergistic [21]. On the other hand, when forced to express *SOX2*, gastric cancer lines were arrested in G1/S transition and undergone apoptosis [14]. Two additional lines of evidence further support that downregulation of *SOX2* may be involved in early stages of gastric carcinogenesis. Downregulation of *SOX2* could be detected in precursor lesions of gastric cancer such as intestinal metaplasia [13] and *Helicobacter pylori *infection, a strong risk factor of gastric cancer, could induce intestinal metaplasia through inhibition of *SOX2* expression [35]. It is possible that *SOX2* also participate in the early carcinogenesis of endometrial cancer via interaction with other risk factors.

## 5. Conclusion

In summary, hypermethylation in association with reduced expression of *SOX2* was demonstrated in endometrial carcinoma. Stem cell transcription factors are likely to play a role in endometrial carcinogenesis.

## Figures and Tables

**Table 1:** Sequences of primers used in *SOX2* methylation-specific PCR.

Primer	Primer sequence (5′ to 3′)	Product size (bp)	Ref.
*SOX2 promoter MSP-M*			
Forward	TGTTTATTTATTTTTTTCGAAAAGGCG	206	[21]
Reverse	GAACCCAACCTCGCTACCGAA		
*SOX2 promoter MSP-U*			
Forward	TGTTTATTTATTTTTTTTGAAAAGGTG	208	[21]
Reverse	CTCAAACCCAACCTCACTACCAA		

**Table 2:** Sequences of primers used in quantitative Real-Time RT-PCR study.

Primer	Primer sequence (5′ to 3′)	Product size (bp)	Ref.
*SOX2*			
Forward	CGAGATAAACATGGCAATCAAAAT	85	[21]
Reverse	AATTCAGCAAGAAGCCTCTCCTT		
GAPDH			
Forward	TCCATGACAACTTTGGTATCGTG	72	[21]
Reverse	ACAGTCTTCTGGGTGGCAGTG		

**Table 3:** Correlation of methylation status of the *SOX2* gene in endometrial carcinomas and normal endometrial tissues.

	Normal endometrial tissue	Endometrial carcinoma	*P* value (chi square)

Status	Frequency (%)	Frequency (%)	
Methylated	1 (8.3%)	27 (37.5%)	.0472
Unmethylated	11 (91.7%)	45 (62.5%)	
Total	12	72	

**Table 4:** Correlation of *SOX2* methylation status with clinicopathological features in endometrial cancers.

Clinicopathological Features	Presence of methylated alleles	Absence of methylated alleles	*P*-value
	Cases (%)	Cases (%)	
Histological type			
Endometrioid	16 (66.7)	32 (84.2)	.108
Serous/CCC	8 (33.3)	6 (15.8)	

Grade			
Low (1)	7 (29.2)	15 (39.5)	.409
High (2-3)	17 (70.8)	22 (60.5)	

Stage			
I	19 (79.2)	32 (84.2)	.613
II–IV	5 (20.8)	6 (15.8)	

Myometrial invasion			
<1/2	8 (72.7)	27 (81.8)	.517
≥1/2	3 (27.3)	6 (18.2)	

Vascular invasion			
Negative	12 (80.0)	27 (73.0)	.596
Positive	3 (20.0)	10 (27.0)	

Involving cervix			
Negative	13 (86.7)	34 (91.9)	.962
Positive	2 (13.3)	3 (8.1)	

Age			
<45	9 (36.0)	10 (26.3)	.413
≥45	16 (64.0)	28 (73.7)	
